# Editorial for the Special Issue RF MEMS and Microsystems: From Reconfigurable Meta-Surfaces to Advanced MIMO and Miniature Antenna Systems

**DOI:** 10.3390/mi17091045

**Published:** 2026-09-01

**Authors:** Marco A. Panduro

**Affiliations:** CICESE Research Center, Km. 107, Carretera Tijuana-Ensenada 3918, Ensenada 22860, Baja California, Mexico; mpanduro@cicese.edu.mx; Tel.: +52-(646)-175-0555; Fax: +52-(646)-175-0554

The relentless expansion of modern wireless communications, high-resolution radar imaging, and decentralized mobile networks demands unprecedented adaptability, extreme compactness, and superior radiation efficiency from electromagnetic systems [1,2]. As global industrial standards push beyond conventional sub-6 GHz allocations into millimeter-wave (mm-Wave) frequencies to achieve multi-gigabit throughputs, traditional antenna paradigms encounter severe physical and operational bottlenecks [3]. At these higher frequencies, path loss scales quadratically according to Friis’ transmission law, forcing engineers to rely on high-gain arrays that must remain ultra-compact to fit within the stringent real estate of consumer hardware [4].

The implementation of these high-density architectures introduces a major technical hurdle: severe spatial constraints that degrade performance. When multiple radiating elements are packed into sub-wavelength footprints, the integration of high-density multi-port arrays often leads to degrading mutual coupling [5]. This electromagnetic coupling distorts individual element radiation patterns, alters expected input impedances, and triggers severe scan blindness in phased arrays [6].

Concurrently, the physical volume required for multi-band operations directly clashes with the aggressive miniaturization demands of consumer electronics and next-generation Internet of Things (IoT) hardware. Standard reactive loading and multi-branch architectures traditionally used to achieve multi-resonance inherently degrade radiation efficiency and narrow the operational impedance bandwidth, a limitation governed by the fundamental Chu–Wheeler physical size limits [7].

To circumvent these fundamental boundaries, contemporary microwave engineering has progressively transitioned away from passive, static geometries toward intelligent, modal-driven architectures and electronically reconfigurable structures [8]. By actively manipulating the surface currents and localized boundary conditions of a radiator, modern structures can dynamically adapt their frequency, polarization, and radiation beams in real time.

This editorial focuses on six research papers published in this issue of *Micromachines*. These papers describe techniques ranging from advanced mathematical modeling (Contribution 1) and Characteristic Mode Analysis (CMA) (Contribution 2) to active or phase-tailored meta-surfaces (Contributions 3–5) and multi-mode decoupling mechanisms (Contribution 6).

Article 1 addresses two ultra-wideband monopoles in a colinear structure for application in remote terrestrial communication systems. The antennas consist of a loaded monopole antenna with a hat and an elevated loaded monopole antenna located in the upper position. All lumped loads are modeled as linear frequency-dependent components to approximate the practical component property for achieving ultra-wideband characteristics, since the constant value property of a component is only present in a relatively narrow band. With the optimized load parameters, one antenna covers 30–750 MHz with a VSWR < 3.5 and the other one covers 800 MHz–3000 MHz with a VSWR < 2.5, which are promising results for terrestrial omnidirectional applications.

Article 2 contributes research of how shaping the ground plane of a wireless device can enhance bandwidth and antenna efficiency, specifically in low-frequency bands of 824–960 MHz, a common frequency band used in IoT where transmitting a small amount of data provides long battery life. Article 2 shows that by adding a slot in the ground plane, the current distribution is enlarged, which enables the excitation of its fundamental mode and, consequently, enhances the bandwidth and antenna efficiency by 2 dB. This approach is assessed using three different printed circuit boards (PCBs) that aim to characterize different factors of IoT devices.

Article 3 contributes the impact of radomization on the radiation pattern of a millimeter-wave antenna for an ETA system utilizing synthetic aperture radar (SAR) with special emphasis placed on the phase shift across both the beamwidth and the bandwidth, rather than the amplitude. Three different radomization approaches, including one based on metasurfaces, are evaluated for a radar antenna operating within the 24.05–24.25 GHz frequency range. The metasurface-based radome provides the best results among the radomization options analyzed.

Article 4 contributes a reconfigurable frequency-selective surface (R-FSS) designed to dynamically switch between WLAN, WiMAX, and sub-6 GHz band frequencies. The electronic switching mechanism of this R-FSS is controlled in real-time using PIN-diodes. Depending on the activation state of these diodes, the structure operates in three distinct modes. This reconfigurability allows the proposed structure to operate across WLAN, sub-6 GHz, and WiMAX frequency ranges either with two narrow stopbands or with a single-wide stopband, while providing polarization selectivity for frequency-selective applications.

Article 5 offers multi-beam and beam-scanning antennas that enable extensive communication coverage while mitigating multipath fading and enhancing spectrum utilization efficiency. Article 5 presents a transmissive meta-surface antenna design, which utilizes a microstrip square-ring patch antenna with four feed ports as the excitation source. A 7 × 7 square patch meta-surface is positioned above the feed source, facilitating the generation of four independently steerable beams by switching activation among the four feed ports. This approach provides the benefits of low profile and low cost with flexible manipulation of electromagnetic wave radiation, thereby providing a novel methodology for designing multi-beam communication antennas.

Article 6 addresses the consumer-electronics bottleneck by developing an ultra-compact, tri-band 4×4 MIMO antenna system. The authors utilized a sophisticated multi-mode excitation structure, allowing an incredibly small physical element to resonate efficiently across all three WiFi 7 frequency allocations. Furthermore, they introduced specialized decoupling slots and isolated circuit paths within the ground plane. This suppressed mutual coupling below −15 dB and drove the Envelope Correlation Coefficient (ECC) far below standard thresholds. This work contributes a fully realizable, high-isolation antenna framework that unlocks the true data-rate potential of WiFi 7 in ultra-slim hardware form factors.

The six articles compiled in this issue highlight a clear paradigm shift in RF, microwave, and millimeter-wave engineering. Rather than treating physical size, mutual coupling, and material phase distortion as insurmountable limitations, these researchers have leveraged advanced modal mathematics, dynamic semiconductor integration, and pixelated meta-surfaces to bypass conventional boundaries. Collectively, these works lay down a robust, highly innovative technical foundation that will directly fuel the development of faster, smaller, and vastly more intelligent global communication networks.
